# Advanced Composites Manufacturing and Plastics Processing

**DOI:** 10.3390/ma18071670

**Published:** 2025-04-05

**Authors:** Patricia Krawczak, Ludwig Cardon

**Affiliations:** 1 Centre for Materials and Processes, IMT Nord Europe, Institut Mines-Télécom, University of Lille, 941 Rue Charles Bourseul, 59508 Douai, France; 2 Centre for Polymer and Material Technologies, Department of Materials, Textiles and Chemical Engineering, Ghent University, Technologiepark 130, 9052 Zwijnaarde, Belgium

## 1. Introduction

Environmental and energy concerns and digitalization are currently profoundly reshaping the plastics and composites industry. Manufacturing processes and systems evolve accordingly in order to cost-effectively produce high-performance, high-quality, lightweight, and multifunctional parts with a reduced carbon footprint. All composites manufacturing and polymer processing technologies are concerned with this trend: liquid composite molding (e.g., resin transfer molding and resin infusion/vacuum infusion), automated lay-up (e.g., automated fiber placement and automated tape laying), filament winding, prepreg technology, pultrusion, autoclave, compression molding, film stacking, additive manufacturing/3D printing, injection molding, over-molding/back-molding, extrusion, blow molding, thermoforming, rotational molding, foaming, coating, preforming of textile reinforcement, joining/welding, and mold technologies (i.e., mold making and design).

This topic gathers 25 original research articles and one technical note on the latest advances in composites manufacturing and plastics processing. These contributions address new process developments, modeling/simulation, monitoring/control, and performance or application issues, with either experimental or numerical approaches. All types of polymers (thermoplastics, thermosets, and elastomers) and fibers/fillers (glass, carbon, ceramic, mineral, and vegetal) are concerned, whether they come from recycled, bio-based, or fossil feedstocks.

## 2. Advanced Composites Manufacturing

### 2.1. Coupling/Hybridization of Processes

Hirsch et al. [1] have optimized the manufacturing of hybrid fiber-reinforced polyamide composite structures by coupling fused granular fabrication (FGF)—a large format additive manufacturing (LFAM) technology that focuses on cost-effective granulate-based manufacturing by eliminating the need for semi-finished filaments—and automated tape laying (ATL). A significant improvement in the flexural stiffness and strength of the manufactured FGF structures was observed by hybridization with 60% glass fiber-reinforced polyamide 6 unidirectional tapes.

Atzler et al. [2] have investigated the overprinting of continuous fiber-reinforced laminates, aiming at producing high-performance, functional structures by means of a hybrid process combining fused granular fabrication (FGF) with automated fiber placement (AFP), where an additively manufactured structure is bonded in situ onto a thermoplastic laminate. The authors have proposed a workflow for the compensation of substrate defects when overprinting in extrusion-based processes. The novel process flow uses a 3D scan of a laminate to adjust the geometry of the additively manufactured structure in order to achieve a constant layer height in the 3D print and, thus, constant mechanical properties.

Kizak et al. [3] have combined additive manufacturing and injection molding and focused on the interfacial fusion bonding of hybrid additively manufactured components made of polycarbonate (neat, with glass or carbon fiber reinforcement) under torsional loading. The effect of various surface treatments (sandpaper, sandblasting, plasma) on injection-molded parts and the influence of different build chamber temperatures during additive manufacturing on torsional strength were specifically examined. The findings emphasize the critical role of surface treatment for the injection-molded components before additive manufacturing. Also, the torsional strength could be increased by up to 87% by actively heating the build chamber.

Picard et al. [4] have explored the manufacturing of cost-effective and customized composites by strategically placing carbon fiber-reinforced thermoplastics in multi-material designs. A model for the simultaneous processing of non-reinforced and reinforced thermoplastic layers, also known as insert overmolding, was developed with the aim of identifying essential parameters to minimize insert flow and ensure desired fiber orientation and positional integrity. A process window for tailored reinforcements in overmolding processes can be easily defined that way.

Ebert et al. [5] have addressed the challenging issue of the functionalization of highly filled continuous glass fiber-reinforced thermoplastic pultruded profiles by welding. For this purpose, the classic hot-tool welding process was adopted, and an infrared emitter with line focus was integrated for heating the polyamide 6 thermoplastic profiles. Despite a very high fiber content of 70 vol.%, a strong welded joint between the unreinforced tension rod and the pultruded plate could be achieved.

### 2.2. Automated Fiber, Yarn, or Tape Placement and Additive Manufacturing

Legenstein and Fauster [6] have studied the effect of flashlamp heating system parameters in order to maximize the wedge peel strength of thermoplastic carbon fiber tape in the automated tape placement (ATP) process. Flashlamp heating systems are rather new in this field of application and offer high energy density with low safety requirements and moderate costs compared to laser-assisted automated tape placement systems.

Shi et al. [7] have proposed a peel test method to characterize the decay law of prepreg tape tack, a key factor affecting the automatic tape laying process, at different temperatures. A new tack test device was designed, and the decay rate of prepreg tack at different temperatures was tested. Based on the experimental results, a prepreg tack decay model was also proposed. Another achievement of the study is a new statistical unit for prepreg tack, which can establish the relationship between the tack of prepreg and its remaining storage time and reduce prepreg management costs.

Knoch et al. [8] have implemented a novel approach in modeling the system limits of a braked, high-speed direct yarn-laying process with in situ impregnation of carbon fiber heavy tows as textile reinforcements for concrete parts. In particular, the yarn spool overrun after the robot has come to a standstill was investigated and modeled using physical equations, taking into account the travel speed, acceleration of the robot, and braking force of the spool brake. Furthermore, models for robot braking time, carbon spool diameter, and spool mass were developed.

Consul et al. [9] have explored large format additive manufacturing (LFAM) with carbon fiber-reinforced polyaryletherketones (PAEK), particularly a slow crystallizing grade, and investigated how extrusion parameters (including line width, layer height, layer time, and extrusion temperature) affect the mechanical properties of the printed parts. Thermal history during printing was monitored using thermocouples and infrared cameras. The study suggests aggregated metrics, enthalpy deposition rate, and shear rate under the nozzle that should be maximized to enhance mechanical performance. Interestingly, the common practice of setting fixed layer times does not ensure repeatable part quality.

Baddour et al. [10] have designed prepregnation and fused filament fabrication parameters for recycled PP- and PA-based continuous carbon fiber (cCF) composites. Plasma treatment of the cCF was also explored, as was the annealing of the produced parts to enhance the flexural properties. Eventually, overall guidelines were formulated for the successful production of cCF-based composites.

Antolin-Urbaneja et al. [11] have addressed the screw-extrusion of carbon fiber-reinforced polyamide in pellet form in order to design and 3D-print aeronautical mold preforms, based on an extensive experimental characterization campaign and a multiphysics computational guidance. The designed mold, around 90% lighter than the original invar design, was numerically proven to fulfill thermal and mechanical requirements with high performance.

### 2.3. LFT Compounding

Aiming at improving the mechanical properties of injection-molded long fiber thermoplastic pellets produced by a thermoplastic pultrusion process, Tipboonsri and Memon [12] have added polypropylene-graft-maleic anhydride (PP-g-MAH) as a coupling agent during the injection molding process. A 4 wt.% concentration of PP-g-MAH was found to be optimal to reach the highest tensile, flexural, and impact strengths.

### 2.4. Fibers and Fabrics Production

Semitekolos et al. [13] have developed a pilot-scale fiber sizing line, including its initial design and installation, operational phases, and optimization of key process parameters. Furthermore, a range of sizing solutions was investigated and formulated, exploring carbon-based nanomaterial types with different surface functionalization and concentrations to evaluate their impact on the surface morphology and mechanical properties of carbon fibers. The incorporation of nanomaterials, specifically N2-plasma-functionalized carbon nanotubes and few-layer graphene, has demonstrated notable improvements (90% increase) in interfacial shear properties.

Ivars et al. [14] have investigated the impact of carding and blending recycled carbon fibers (rCF) with crimped thermoplastic polypropylene (PP) fibers on the mechanical properties of rCF using a Weibull statistical approach. Overall, carding rCF with PP fibers helps in the mechanical property uniformity of the resulting carded webs without compromising tensile performance. The work also highlights the potential of the carding process with or without thermoplastic fibers to efficiently realign and give continuity to discontinuous recycled carbon fibers.

### 2.5. Manufacturing of Sustainable Bio-Based Composites

Pisupati et al. [15] have elucidated the influence of the cooling rate, and thus of the crystallinity, on the bending and impact properties of compression-molded non-woven flax/polylactic acid (PLA) biocomposites with different flax fiber contents. An improvement of 25% and 100% in flexural modulus was observed for the composites with 40 wt.% and 50 wt.% of flax fiber, respectively, when subjected to a lower cooling rate. This makes such flax/PLA composites promising for semi-structural applications, providing a sustainable alternative with enhanced lightweight performance.

Li et al. [16] have compared the physical properties of coconut fiber-reinforced polypropylene composite materials, produced through a hybrid injection molding process and a layered hot-pressing process. The layered hot-pressed composite material exhibited optimal comprehensive mechanical performance and could be applied to airbag covers in the field of automotive safety, as confirmed by the modeling and finite-element simulation carried out.

### 2.6. Laminated Glass/Polymer Composites

Jeong et al. [17] have proposed an equivalent model of polyvinyl butyral (PVB) laminated glass to simulate the head injury criterion (HIC) when a pedestrian collides with a tram rail vehicle. Whereas the traditional PLC model replicates the multi-layer structure of PVB-laminated glass, including tempered glass and PVB film, the new simplified equivalent model considers tempered glass and PVB film as a single shell element layer, incorporating the multi-layer characteristics via integration points. As analytical equivalency with the PLC model is maintained, the equivalent model produces results comparable to the PLC model while significantly reducing analysis time from 8 h to 1 h under the same hardware and software conditions.

## 3. Machining, Milling, and Drilling of Polymers, Composites, and Multi-Material Laminates

Nargis et al. [18] have optimized the drilling quality of unidirectional carbon fiber-reinforced polymer composites, as well as hybrid Al_2_O_3_ alumina and hybrid SiC silicon carbide nanocomposites, through an experimental exploration using step, core, and twist drills. Response surface methodology (RSM) and statistical tools were used to analyze the surface roughness of the hole, and two machine learning models—artificial neural network (ANN) and random forest (RF)—were used for predictive analysis. Both models have shown robust predictive capabilities, with RF demonstrating superior performance over ANN and RSM.

Bolar et al. [19] have analyzed the performance of helical milling and drilling operations while machining carbon fiber-reinforced aluminum laminates. Indeed, being a difficult-to-cut material, fiber/metal laminates often pose challenges during conventional drilling and require judicious selection of machining parameters to ensure defect-free laminates. Helical milling appears to be a promising technique for producing good-quality holes, having a lower surface roughness than that achieved by conventional drilling.

Wu et al. [20] have investigated the machinability and surface properties of cryogenically cooled poly(methyl methacrylate) machined via single-point diamond turning (SPDT). Cryogenic machining was shown to be useful for improving the form accuracy and reducing the surface roughness of PMMA.

## 4. Polymer Processing and Compounding

### 4.1. Functionalization

Andrade-Guel et al. [21] have elaborated by melt-blowing functional technical non-woven fabrics from polymer nanocomposites made of polyamide 6 and amine-modified nanoclay having adsorbent properties of toxins and dyes and also showing an antibacterial behavior.

Alkarri et al. [22] have paid attention to the antimicrobial, thermal, mechanical, and gas barrier properties of linear low-density polyethylene extrusion blow-molded bottles. New methods of incorporating antimicrobial particles into the bottles were developed. The antimicrobial particles were thermally embossed on the external surface of the bottle through two particle deposition approaches (spray and powder) over the mold cavity. Both deposition approaches have led to a significant enhancement in antimicrobial activity, as well as barrier properties, while maintaining thermal and mechanical performance.

Prihandana et al. [23] have investigated the influence of activated carbon (AC) particle size on the properties and performance of polysulfone composite membranes for protein separation. The polymeric composite membranes were fabricated via the phase-inversion method, employing water as the coagulant and using variable loadings of AC particle sizes. The impact of the AC powder particle sizes on membrane morphology, water contact angle, porosity, average pore size, molecular weight cutoff, pure water flux, and protein rejection was examined. On this basis, recommendations could be provided for the selection of AC particle sizes for protein separation in conjunction with polysulfone ultrafiltration membranes.

Al Mais et al. [24] have highlighted the importance of diverse forms, e.g., morphology and particle size, and concentrations of graphitic carbon nitride (g-C_3_N_4_) as strengthening elements in thermosetting epoxy resin-based composites. Optimal mechanical properties were achieved with a 0.5 wt.% bulk g-C_3_N_4_ filler, enhancing tensile strength by 14%. An increased toughness, no significant impact on the degradation temperature, a 17% increase in glass transition temperature, and an improvement in thermal breakdown up to 600 °C were also noticed.

### 4.2. Plastics Recycling

Gall et al. [25] have compared end-of-life vehicle (ELV) and packaging-based recyclates as components in polypropylene (PP)-based compounds designed for automotive applications. Two ELV recyclate grades based on bumper recycling were analyzed in comparison to a packaging-based post-consumer recyclate (PCR) and compounded with virgin PP base material and mineral reinforcement. While the targeted mechanical properties could nearly be reached, further development work is still necessary to improve the flowability of the PCR compounds.

Through a twin-screw extrusion process, Kharmoudi et al. [26] have formulated blends based on post-consumer recycled high-density and high-molecular-weight polyethylene used for manufacturing rainwater drainage pipes and investigated the effect of graphene addition on mechanical and thermal behavior. Adding low amounts of graphene to recycled polyethylene blends could be an interesting alternative to ensure good mechanical properties and a resistance to crack propagation comparable to virgin polyethylene compounds.
